# Point prevalence of motor neuropathy in children and adolescents with type 1 diabetes mellitus

**DOI:** 10.1007/s40200-026-01865-z

**Published:** 2026-02-26

**Authors:** Joana Helena Bourbon Lopes, Jacinta Fonseca, Fernando Silveira, Cíntia Castro-Correia

**Affiliations:** 1 Faculdade de Medicina da Universidade do Porto (FMUP), Alameda Prof. Hernâni Monteiro, Porto, 4200 − 319 Portugal; 2 Serviço de Pediatria, ULS São João, Departamento de Ginecologia- Obstetrícia e Pediatria, Faculdade de Medicina da Universidade do Porto (FMUP), Alameda Prof. Hernãni Monteiro, Porto, 4200 − 319 Portugal; 3 Unidade de Neurofisiologia, Serviço de Neurologia, ULS São João, Alameda Prof. Hernâni Monteiro, Porto, 4200 − 319 Portugal; 4 Serviço de Pediatria, ULS São João, Departamento de Ginecologia- Obstetrícia e Pediatria, Faculdade de Medicina da Universidade do Porto (FMUP), Alameda Prof. Hernâni Monteiro, Porto, 4200 − 319 Portugal

**Keywords:** Motor neuropathy, Nerve conduction studies, Type I diabetes mellitus, Paediatrics

## Abstract

**Purpose:**

Our objective is to conduct a screening for motor neuropathy in children and adolescents with type 1 diabetes to assess its point prevalence and to analyse potential risk factors associated with any positive motor neuropathy diagnosis.

**Methods:**

This is a cross-sectional study involving children aged 12 to 18 years who have been diagnosed with diabetes for five or more years and are receiving treatment with an insulin pump. All participants underwent a neurological examination and were questioned about symptoms of neuropathy. A nerve conduction study was conducted to evaluate the median, ulnar, common peroneal, and tibial motor nerves. Sensory nerves were also examined. The F-wave response of the tibial nerve was analysed, and needle electromyography was performed on a proximal and distal muscle of the lower limb.

**Results:**

A total of 29 children completed the study (mean age: 15.34 ± 1.56 years; mean duration of diabetes: 11.93 ± 2.84 years; HbA1c levels: 7.50 ± 1.17%). Results were normal, indicating adequate motor nerve integration and excluding the presence of motor neuropathy as well as peripheral neuropathy, even at subclinical level.

**Conclusion:**

In our studied population, which receives tight monitoring and support for diabetes management, using nerve conduction studies to detect early subclinical motor neuropathy shows no clear benefit. This finding was consistent even among individuals with poor metabolic control, altered albumin/creatinine ratio, and diabetes duration over 10 years, with no abnormalities observed. We recommend following the latest guidelines provided by the American Diabetes Association (ADA).

**Supplementary Information:**

The online version contains supplementary material available at 10.1007/s40200-026-01865-z.

## Background

### Type 1 diabetes(T1DM)

Type 1 diabetes mellitus (T1DM) is a chronic autoimmune disorder characterized by insulitis, an inflammatory lesion caused by autoreactive T-cells that infiltrate the islets, leading to the destruction of β-cells and ultimately resulting in insulin deficiency [1–3]. The most common symptoms resulting from hyperglycaemia include polyuria, weight loss, polydipsia, and, in some cases, diabetic ketoacidosis. Diagnosis is based on clinical symptoms and glucose monitoring [4–6]. It is the most common chronic endocrine disease among the paediatric population [7].

### Diabetic peripheral neuropathy

Prolonged T1DM can cause early microvascular complications like retinopathy, nephropathy, and neuropathy, while macrovascular consequences like arteriosclerosis tend to occur later [8]. Diabetic neuropathies affect the nervous system, causing various symptoms due to abnormalities in sensory, motor, and autonomic nerve fibres, which are classified based on clinical features [9].

Chronic sensorimotor distal symmetric polyneuropathy (DSPN) and autonomic neuropathy are the two most common types of neuropathies [9–11]. Diabetic peripheral neuropathy (DPN) falls under the DSPN category. DPN is defined as symptoms or signs of peripheral nerve dysfunction in individuals with diabetes, excluding other causes [11]. It is a common complication that can lead to significant disability, reduced quality of life, and a substantial economic burden [12].

It is estimated that up to 50% of individuals with long-term diabetes may be affected by this complication [11, 13]. This percentage could rise to 100% when more precise diagnostic methods, such as nerve conduction studies, are used [11]. The prevalence of these complications is likely underestimated, as they often remain subclinical in younger age groups [9, 11].

Although younger age at onset, longer duration of diabetes, and a history of diabetic complications are recognised risk factors, the primary driver is chronic hyperglycemia [9, 11]. This sustained metabolic imbalance leads to the formation of advanced glycation end products and activation of polyol, glycolysis and hexamine biological pathways which trigger oxidative stress at the cellular level, resulting in vascular and neuronal damage. Therefore, it is crucial to maintain good metabolic control [11].

DPN affects both myelinated and unmyelinated nerve fibres. Pain mainly results from damage to thin, unmyelinated fibres, while impairment of large, myelinated fibres can lead to gait instability, increasing fall risk [2]. Common symptoms like numbness, tingling, allodynia, and a shock-like sensation, indicate somatic involvement [11].

According to the American Diabetes Association (ADA), screening for DPN is recommended five years after diagnosis in type 1 diabetic patients. The frequency of this screening should be annual or semestral and must include clinical history and a physical examination [13].

### Motor neuropathy and nerve conduction studies

Motor involvement in DPN is often overlooked compared to sensory dysfunction [14]. However, motor impairment increases fall risk, affects gait and balance, and can lead to foot deformities like hammer toes, contributing to chronic irritation and ulceration [14, 15].

Multiple studies using nerve conduction studies (NCS) have shown the ability to detect subclinical motor involvement, revealing reduced motor nerve conduction velocity (MNCV) and amplitude, indicative of nerve deterioration. Early MNCV reduction is linked to nodal dysfunction, axonal swelling, oxidative stress, and metabolic disturbances. Over time, axonal atrophy and segmental demyelination leads to conduction blocks. When detected early, these conditions can be reversed with proper metabolic control, including insulin administration; however, after a few months, these abnormalities become irreversible [14].

Studies emphasize the importance of NCS in detecting early motor dysfunction, revealing a high prevalence of subclinical neuropathy in diabetic patients and indicating that motor involvement is more frequent than previously recognised [10, 14, 16]. Longitudinal research demonstrates a significant increase in DPN over time, with subclinical cases nearly doubling after several years, underscoring the progressive nature of the condition [12].

These findings substantiate the hypothesis that NCS provide significant advantages for early screening and detection of motor neuropathy. By identifying nerve dysfunction before clinical symptoms emerge, these tests enable earlier interventions, potentially slowing progression and preventing severe motor complications. Although subclinical nerve function abnormalities may not directly predict the onset of clinical neuropathy, certain changes in nerve function indicate damage that, when combined with other local injuries, may become clinically significant [17].

Despite extensive research on peripheral neuropathy in type 1 diabetes, the pattern of nerve involvement remains controversial. The frequent involvement of the common peroneal nerve in some studies further suggests a targeted approach in screening protocols, making NCS a valuable tool beyond standard neurological exams [8, 10].

This method can be time-consuming, expensive, and uncomfortable for many paediatric patients. However, it is less dependent on patient cooperation, making it less subjective. In children and adolescents with T1DM, early signs of neuropathy are often minimal or absent, making clinical exams less reliable in this group [9, 18]. Nerve conduction studies can therefore be a more effective diagnostic tool, as they can detect the condition at a subclinical stage.

### Objectives

The main objective was to evaluate the point prevalence of motor neuropathy in children and adolescents with a 5-year or more diagnosis of type 1 diabetes using nerve conduction studies. Various biochemical and clinical parameters of each individual were analysed to assess metabolic control. Subsequently, to identify potential factors associated with an increased risk of motor neuropathy, we planned to evaluate motor neuropathy diagnoses in relation to these parameters.

## Methods

This cross-sectional analytical study was conducted at the paediatric endocrinology department of the Centro Hospitalar Universitário S. João (CHUSJ), from September 2024 to March 2025.

We reviewed all the children who attended the semester evaluation consultation for their T1DM diagnosis between September 2024 and January 2025. Among them, we found a total of 42 children who met our inclusion criteria: they were aged between 10 and 18 years, had been diagnosed with T1DM for over five years, and were being treated with an insulin pump. We then excluded any children who had been diagnosed with neuropathy due to causes other than T1DM, such as infections, connective tissue diseases, medications, or nutritional deficiencies. We also excluded children with a family history of hereditary neuropathy, those with types of diabetes other than type 1, and children using medications that could potentially affect peripheral nerve function.

The total number of participants was determined based on a review of similar studies, the total number of children monitored at CHUSJ, and practical considerations such as time constraints and the availability required to conduct both NCS and neurological examinations.

The study was approved by the Ethics committee of CHUSJ. Written informed consent was obtained from patients and their parents. Participants had the right to withdraw from the study at any time. Confidentiality and the well-being of patients were prioritized throughout the study.

The enrolled children underwent a standardized and systematic summary neurological examination which included ankle, patellar, bicipital and tricipital reflex, assessment of muscular force, vibration and cutaneous sensation. All evaluations were conducted by a single individual to eliminate interobserver bias, thereby reducing variability in the application and interpretation of the neurological examination.

Vibration was evaluated using a 128 Hz tuning fork placed on the dorsum of the great toe, just proximal to the nail bed and scored as (= 1) if present (= 0) if absent.

The analysis of cutaneous touch sensation was conducted using the 10 g Semmes-Weinstein monofilament on the plantar surface of the great toe and the bases of the first and fifth metatarsals on both feet. The monofilament was applied perpendicularly to intact skin with enough pressure to cause it to bend, maintaining contact for no longer than two seconds. The individuals were with their eyes closed and described whether they felt the pressure and where they felt it. The test was assessed in three specific sites, making three applications at each site while alternating between actual touches and simulated ones. Cutaneous sensation was considered present if at least two out of three responses were correct for each site, with a score of (= 1) if present and (= 0) if absent [19].

The assessment of muscular force involved extending and flexing the ankle, knee, and elbow, which was then graded using the Medical Research Council (MRC) scale from zero to five. Muscle power was classified as follows: normal for grades 4–5, reduced for grade 3, and severely reduced for grades 0–2.

The reflexes were evaluated using a reflex hammer and scored as normal (= 0), present with reinforcement (= 1) and decreased or absent (= 2).

Following the neurological summary examination, participants were inquired about the presence of various symptoms suggestive of neuropathy, including cramps, numbness, tingling, burning sensations, electric shock-like sensations, instability while walking, fatigue, hyperalgesia, and allodynia. The symptoms were classified as follows: (= 1) if present and (= 0) if absent.

The electrophysiological test was done by the dantec keypoint electromyography machine by a single neurophysiologist who was blinded to the patient´s clinical and medical history. The exam was, in general, well tolerated by the children and adolescents. The temperature was checked each time before placing the electrodes, ensuring it was above 30 °C. When necessary, arms and legs were warmed with hot water. The ground electrode was placed midway between the stimulation and the recording electrodes.

Motor conduction studies were performed bilaterally on the median, ulnar, common peroneal, and tibial motor nerves. Conduction velocity (CV) on the tibial nerve was not measured, as it can be uncomfortable. Instead, we opted to measure the F wave response, which was recorded in both the right and left tibial nerves. This technique has a higher sensitivity as it studies also proximal conduction [20]. F minimal latency (m s) was considered normal if equal to or lower than 55 m s.

Table 1 shows the reference cut-off values considered normal and routinely used in clinical practice. Considering these reference values, the electrophysiological findings were analysed by a neurologist trained in clinical neurophysiology and neuromuscular disorders. They were determined through a literature review by neurophysiology specialists, based on the American Association of Neuromuscular & Electrodiagnostic Medicine (AANEM) guidelines and adapted to the Portuguese population [21].

**Table 1:** Reference values for nerve conduction studies that are considered normal

Nerve	Stimuli	Response registration	Distal Latency (m s)	Amplitude (M v)	Velocity (m s⁻¹)	Distance (cm)
Peroneal	Ankle	Extensor digitorum brevis	≤ 6.0	≥ 2.0*	-	-
		Extensor digitorum brevis	-	-	≥ 40	-
Tibial	Posterior to the medial malleolus	Hallux abductor	≤ 6.0	≥ 4.0*	-	-
Medianus	wrist	Abductor pollicis brevis	≤ 4.2	≥ 4.0	≥ 50	8.0
Ulnaris	Wrist	Abductor digiti minimi	≤ 3,6	≥ 6,0	-	8–12
	Below elbow	Abductor digiti minimi	-	-	≥ 50	-

*Compare with the contralateral

Needle electromyography (EMG) was conducted on all individuals for the right peroneus tertius and right vastus medialis, as this test shows greater sensitivity for axonal motor damage. The reduction in the amplitude of the compound muscle action potential (CMAP), with either normal or slightly decreased CV, or a modest increase in distal motor latency (DML) can support axonal damage [22].

Primary demyelination can be considered when there is a marked reduction in motor CV or grossly increased DMLs or minimum F-wave latencies [22].

Data were collected and analysed, including age, gender, height, weight, body mass index (BMI), duration of diabetes, haemoglobin A1c (HbA1c), fasting plasma glucose, lipid profile (HDL, LDL, total cholesterol, triglycerides), time in range (TIR) percentage, coefficient of variation, vitamin D levels, urea, creatinine, albuminuria and the spot albumin-to-creatinine ratio. Metabolic control was considered good if HbA1c levels were between 6.5% and 7.5%, moderate if between 7.6% and 9.0% and poor if above 9.0% [8].

### Statistical Analysis

Data are presented as arithmetic means ± standard deviations (SD). Three missing conduction velocity values were imputed using the mean after confirming a normal distribution (bolt in Table S1- see Online Resource 1). This preserved the sample size. Two individuals had clinical analyses performed privately, with only confirmation of normal results available. One individual lacked TIR (%) and variation coefficient (%) due to the absence of FreeStyle Libre monitoring (hyphen in Table S1- see Online Resource 1).

## Results

**Fig. 1:**
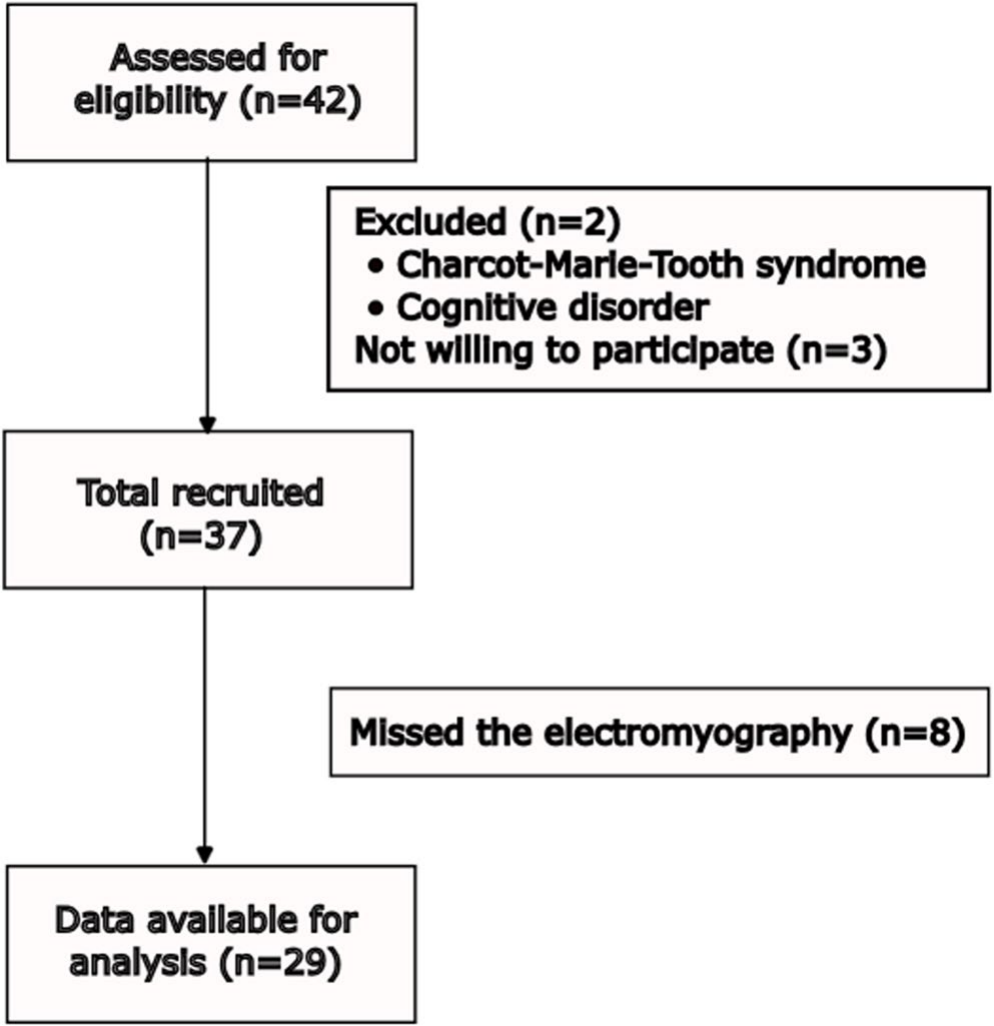
Flow diagram for participant recruitment

Out of the 42 individuals assessed for eligibility, 2 were initially excluded from the study: one had Charcot-Marie-Tooth syndrome and the other had a cognitive disorder. Additionally, 3 individuals declined to participate. Ultimately, a total of 29 participants completed the study (Fig. 1).

Of the 37 individuals who initially agreed to participate, a total of 8 did not attend the scheduled electromyography session. Some individuals gave no reason for their absence, while others were hesitant to miss classes solely for the purpose of the exam. Due to time constraints and scheduling logistics with the neurophysiology doctor, it was difficult to arrange the exam on the days when they had other appointments. If it had been possible, we would have achieved better matriculation.

The mean age of the patients was 15.34 ± 1.56 years, 34.48% were female and 65.52% were male. The mean age duration of T1DM was 11,93 ± 2,84 years. The biochemical characteristics of the patients are shown in Table 2. The HbA1c was 7.50 ± 1.17%, with 37% having values of HbA1c above 7.5%. In addition, 68% had a time in range (%) less than 70% and 61% had a coefficient of variation (%) greater than 36%. None of our patients had retinopathy, and only one individual showed an altered albumin/creatinine ratio.

**Table 2:** Profile of biochemical parameters in study participants

Parameters	Cases (%) *n* = 29
1. BMI (Percentile)	
< P3 (Underweight)	0 (0%)
P3 - P85 (Normal)	21 (72%)
P85 - P97 (Excess weight)	7 (24%)
> P97 (obesity)	1 (3%)
2. Fasting plasma glucose (mg dL⁻¹) (178 ± 40.86)	
< 100 mg dL⁻¹	1 (4%)
100–125 mg dL⁻¹	1 (4%)
126–200 mg dL⁻¹	19 (66%)
> 200 mg dL⁻¹	8 (28%)
3. HbA1c (%) (59 ± 13 mmol/mol (7.5 ± 1.2%))	
< 48 mmol/mol (< 6.5%)	3 (1o%)
48–59 mmol/mol (6.5% -7.5%)	16 (55%)
60–75 mmol/mol (7.6% − 9.0%)	7 (24%)
75 mmol/mol (> 9.0%)	3 (10%)
	Cases (%) *n* = 28
4. Spot urinary albumin/creatinine ratio (mg g ⁻¹) (7.15 ± 9.02)	
< 30 mg g⁻¹ (Normal)	27 (96%)
30–300 mg g⁻¹ (microalbuminuria present)	1 (4%)
Spot urinary Albuminuria (mg L**⁻**¹) (12.60 ± 23.61)	
< 20 mg L**⁻**¹ (Normal)	25 (89%)
> 20mg L**⁻**¹ (Albuminuria)	3 (11%)
5. Urea (mg dL**⁻**¹) (28.57 ± 6.43)	
10.00–50 mg dL**⁻**¹ (Normal)	28 (100%)
> 50 mg dL**⁻**¹	0 (0%)
6. Creatinine (mg dL**⁻**¹) (0.66 ± 0.14)	
0.67 mg dL**⁻**¹- 1.17 mg dL**⁻**¹ (Normal)	15 (54%)
< 0,67 mg dL**⁻**¹ & > 1.17 mg dL**⁻**¹ (Abnormal)	13 (46%)
1. Time in range(%) ( 57 ± 0.19)	
≥ 70% (Normal)	9 (32%)
< 70% (Abnormal)	19 (68%)
2. Variation coefficient (%) (38 ± 0.06)	
≤ 36% (Normal)	11 (39%)
> 36% (Abnormal)	17 (61%)
	Cases *n* = 27
3. Total cholesterol (mg dL**⁻**¹) (152.22 ± 28.10)	
≤ 200 mg dL**⁻**¹ (Normal)	24 (89%)
> 200 mg dL**⁻**¹ (Abnormal)	3 (11%)
4. HDL (mg dL**⁻**¹) (55.65 ± 9.43)	
≥ 35 mg dL**⁻**¹ (Normal)	27 (100%)
< 35 mg dL**⁻**¹ (Abnormal)	0 (0%)
5. LDL-c (mg dL**⁻**¹) (80.96 ± 18.11)	
≤ 130 mg dL**⁻**¹ (Normal)	27 (100%)
> 130 mg dL**⁻**¹ (Abnormal)	0 (0%)
6. Triglycerides (mg dL**⁻**¹) ( 76.07 ± 51.06)	
≤ 170 mg dL**⁻**¹ (Normal)	24 (89%)
> 170 mg dL**⁻**¹ (Anormal)	3 (11%)
Vitamin D (ng mL**⁻**¹) (21.85 ± 8.52)	
> 20 ng mL**⁻**¹ (Normal)	14 (52%)
12–20 ng mL**⁻**¹ (insufficiency)	10 (37%)
< 12 ng mL**⁻**¹ (deficiency)	3 (11%)

HbA1c Hemoglobin A1c or glycated hemoglo 1;HDL High-density; LDL Low-density lipoprotein, BMI body mass index;

During the summary neurological examination, no abnormalities were found, except in four individuals who exhibited decreased patellar and achilles reflexes. Among these, one reported numbness in the legs. No other complaints were noted.

Ultimately, all NCS results were normal. Table 3 summarises the mean values (± SD) for latency, velocity, and amplitude of the peroneal, tibial, median, and ulnar motor nerves. The full dataset is available as Supplementary Material (see Online Resource 1).

**Table 3:** Nerve conduction study results (latency, amplitude, and velocity) presented as mean ± standard deviation

Nerve	Latency (m s)	Amplitude (M v)	Velocity (m s⁻¹)
Left Peroneal	4.11 ± 0.53	5.62 ± 2.17	49.61 ± 3.47
Right Peroneal	3.79 ± 0.35	6.33 ± 2.10	48.69 ± 3.26
Left Tibial	4,11 ± 0,54	11.39 ± 3.65	-
Right Tibial	4.24 ± 0.56	11.47 ± 3.00	-
Left Medianus	3.39 ± 0.33	8.46 ± 1.97	56.90 ± 3.50
Right Medianus	3.39 ± 0.34	9.35 ± 1.93	58.02 ± 2.66
Left Ulnaris	2.56 ± 0.29	8.68 ± 1.29	60.99 ± 4.43
Right Ulnaris	2.66 ± 0.30	8.62 ± 1.55	60.12 ± 4.90

The EMG results were also normal, indicating no axonal damage. Additionally, F minimum latency values were within the normal range (below 55 m s) at 45.59 ± 3.73 m s in the right tibialis and 45.77 ± 3.96 m s in the left tibialis.

## Discussion

Our study found no evidence of subclinical motor neuropathy in any of the participants, suggesting that motor nerve function remained preserved in this population. This contrasts with previous studies that reported a higher prevalence of motor involvement.

Abuelwafaa et al. 2019 studied 50 diabetic patients aged 10–18 years and found that 88% had electrophysiological evidence of peripheral neuropathy, primarily affecting motor function (68.2%) with no cases of pure sensory neuropathy. The most common finding was conduction slowing, particularly in the common peroneal nerve [10]. This finding is noted in other studies, highlighting the significance of motor involvement [16, 23]. They used a stricter conduction velocity threshold of 46.7 m s⁻¹ compared to our 40 m s⁻¹, which may explain their higher prevalence rates [10]. The mean HbA1c level in their population was 11.28 ± 2.75%, compared toour study’s mean of 7.50 ± 1.17%. This indicaes poorer metabolic control, despite a shorter disease duration of 10.21 ± 3.93 years [10].

Singh et al. 2022 study, out of also 50 children aged 8–18 years, 56% exhibiting subclinical neuropathy on NCS, with 40% having pure motor, 2% pure sensory, and 14% mixed motor-sensory neuropathy [8]. The participants had poorer metabolic control with HbA1c of 9.14 ± 2.10% and the higher prevalence may also be due to ethnic and genetic differences [24–26]. Glycaemic variability, including the frequency of hypoglycaemia and hyperglycaemia, and different diagnostic criteria may also contribute. The peroneal nerve was also the most affected, with significant risk factors including poor glycaemic control (HbA1c > 9%) and diabetes duration of over five years [8]. However, this pattern is not consistently observed in other studies, leading to the belief that the development of neuropathy in childhood is neurophysiologically heterogeneous [7, 12, 17].

On the other hand, Walter-Holiner et al. 2018 conducted a cohort study with a 5-year follow-up, in Austria with a total of 38 patients aged 9–18 years [12]. At baseline, the prevalence of diabetic peripheral neuropathy (DPN) diagnosed through neurological examination was 13.2%, while nerve conduction velocity (NCV) testing detected DPN in 31.6%, indicating a high prevalence of subclinical cases. After five years, clinically diagnosed DPN increased to 34.2% (= 0.039), while subclinical DPN rose to 63.2% (= 0.002), with the most significant electrophysiological changes observed in the tibial sensory nerve [12]. Some research indicates that the motor nervous system is more resistant than the sensory nervous system. This difference may be due to anatomy: dorsal root ganglion neurons are outside the blood-brain barrier, while motor neurons are protected within the ventral horn of the spinal cord [7, 27, 28]. While not the main focus of this study, NCSs were also performed on the median, ulnar, and sural sensory nerves. These revealed no abnormalities, thus providing no evidence of diabetic peripheral neuropathy. In addition, they used reference values like ours for conduction velocity in motor nerves. And so given Austria’s advanced healthcare system, the high prevalence is unlikely to be attributable to limited resources or poor metabolic monitoring, as may have been the case in other studies. Their mean HbA1c was 8.1 ± 1.2%, comparable to ours, but with a shorter disease duration of 5.6 ± 3.2 years [12].

There were one or two individual measurements that were close to the reference range, particularly the common peroneal nerve velocity and F minimum latency. Notably, this phenomenon has been observed in taller individuals with no clinical symptoms and no changes in the physical and neurological exam. Studies indicate that taller people typically exhibit lower nerve conduction velocities and higher F wave latencies, which may represent a physiological variation rather than a pathological one [16, 29]. Secondly, there may also be minor technical factors such as electrode positioning which can introduce small discrepancies in motor nerve assessments. These considerations highlight the need for cautious interpretation of borderline values to avoid overestimating pathological findings.

In instances of isolated borderline values, it is crucial to consider the broader context, potential concomitant alterations like altered tibial F waves, altered needle electromyography, changes in physical neurological exam and the individual’s height. For these individuals, a follow-up evaluation is recommended within a five-year period to determine whether there have been any significant changes in the values compared to the initial assessment. If any changes are observed, it may be appropriate to consider the onset of subclinical motor neuropathy.

Consistent and reliable results were achieved through standardized neurological and electrophysiological assessments, which offered a comprehensive and objective evaluation of neuropathy in patients with T1DM. This also ensured comparability across different settings and populations. It is a relatively underexplored area, especially in our Portuguese population contributing to new knowledge to the field.

In addition, by having a single examiner conduct the neurological exam, inter-observer variability was minimized, enhancing the study’s internal validity. However, misinterpretation or subtle variations in reflex evaluations could account for the alterations observed in the four cases. Furthermore, all NCS and EMG assessments were performed by the same highly experienced neurophysiologist, blinded to the patient´s clinical and medical history. This approach minimized, once again, interobserver variability while also reducing observer bias.

Some factors may limit the study´s generalisability. It was conducted in a single centre, CHUSJ, a hospital with experienced physicians and systematic treatment monitoring. This may not fully represent the broader paediatric T1DM population in other hospitals.

This study may be subject to some healthy user bias, as some children or adolescents who chose not to participate had poorer metabolic control and appeared to be less concerned about their health.

There are scarce reference values of electrophysiological results in a healthy paediatric population and so incorporating a healthy control group in a cohort study for comparison would improve external validity. However, it would be difficult to get approval from the ethics committee. Although we did not use a control group for reference and comparison, relying instead on fixed reference values, we could possibly say that our individual and mean ± SD values were very similar to those of control groups used as reference in many studies. There could be some doubt regarding the conduction velocity of the common peroneal nerve, which could eventually show statistical significance if analysed with values from control groups in other studies. Nonetheless, we wouldn’t consider these individual values to be pathological, given the clinical context, the height of the patients, and additional findings such as normal tibial F waves and needle electromyography, which are more sensitive indicators [17, 23, 30]. Still, we did not take this approach because no similar study has been conducted in a Portuguese population with comparable characteristics. Using control groups from studies conducted in other countries could introduce bias due to population differences like genetic background, environmental factors, healthcare systems, and lifestyle that could influence the results.

## Conclusion

This study indicates that, within our population, where patients receive thorough monitoring and support for their diabetes management, there is no discernible benefit of employing nerve conduction studies, like electromyography, for the purpose of diagnosing or detecting subclinical motor neuropathy. Even children and adolescents with poor metabolic control, altered albumin/creatinine ratios, and a long duration of diabetes exceeding 10 years showed no abnormalities in our study. There is still no consensus on the pattern of nerve involvement and histological abnormalities (demyelination or axonal degeneration), so further research is needed in this area to provide more conclusive information on the possibility of primary and secondary prevention and to improve quality of life. The varying prevalence rates presented are mostly due to the different criteria and lack of standardized characterization of DPN [23].

For, now we recommend using the electrophysiological diagnostic method only in accordance with the guidelines established by the American Diabetes Association (ADA). This recommendation applies specifically when clinical features are atypical or when a diagnosis remains uncertain after a comprehensive medical history and basic clinical assessments have been conducted [13].

## Supplementary Information

Below is the link to the electronic supplementary material.

Supplementary Material 1

## Data Availability

All data generated or analysed during this study are included in this published article [and its Supplementary Information Files].

## Abbreviations

ADA: American Diabetes Association
BMI: Body Mass Index
CHUSJ: Centro Hospital Universitário S.João
CMAP: Compound Muscle Action Potential
CV: Conduction Velocity
DPN: Diabetic Peripheral Neuropathy
DML: Distal Motor Latency
DSPN: Distal Symmetric Polyneuropathy
EMG: Needle Electromyography
HbA1c: Haemoglobin A1c
MNCV: Motor Nerve Conduction Velocity
NCS: Nerve conduction studies
SD: Standard Deviations
TIR: Time in Range
T1DM: Type 1 Diabetes Mellitus
