# Supplementary material for: Point prevalence of motor neuropathy in children and adolescents with type 1 diabetes mellitus
Source: J Diabetes Metab Disord. 2026 Feb 26;25(1):88. doi: 10.1007/s40200-026-01865-z (PMC12946334; doi:10.1007/s40200-026-01865-z)
Supplement: Supplementary file 1 — Supplementary Material 1 [file 40200_2026_1865_MOESM1_ESM.pdf]

Supplementary material

Point prevalence of motor neuropathy in children and adolescents with type 1 diabetes mellitus

Journal of diabetes and metabolic disorders

Joana Helena Bourbon Lopes*, Jacinta Fonseca, Fernando Silveira, Cíntia Castro-Correia,

*Corresponding author: Joana Helena de Bourbon Faria Ribeiro Lopes, 6th-year medical student, Faculdade de Medicina da Universidade do Porto (FMUP), Alameda Prof. Hernâni Monteiro, 4200-319 Porto, Portugal; up201908122@up.pt

Table S1-dataset including demographic data, biochemical parameters, results of neurological examinations, and nerve conduction study values.

Sex (F=female M=male)	F	F	F	M	M	M	M	F	M	M	M	M	M	M	M	F	F	M	M	F	F	M	M	M	F	F	M	M	M
DMT1 duration (years)	15	14	14	12	13	15	16	14	10	14	13	12	10	8	13	12	10	15	13	11	14	6	16	13	12	8	8	10	5
Age (years)	17	17	16	17	14	15	17	15	16	15	16	14	12	15	16	14	14	16	15	15	15	12	17	17	17	12	15	16	18
Height (cm)	176	159	165	174	162	175	173	165	175	169	182	167	148	185	170	163	156	172	182	160	155	154	170	187	172	161	166	172	164
Weight (Kg)	77.6	48.0	66.5	60	56.8	60.6	64.4	69.5	63.7	70	69.7	55.5	41.1	65.9	61.5	69.1	55.7	54.9	64.6	54.5	57.6	41.2	64.7	81.8	92.1	42.2	56.8	70.5	70.1
BMI (kg m^{-2})	25.2	19.1	24.4	19.8	21.5	19.9	21.6	25.6	20.9	24.4	21.0	19.9	18.8	19.3	21.3	26.2	23.0	18.5	19.6	21.4	24.0	17.4	22.3	23.4	31.1	16.4	19.8	23.8	26.2
HbA1c (IFCC mmol/mol (NGSP %))	76 (9.1)	58 (7.5)	50(6.7)	57 (7.4)	57 (7.4)	37 (5.5)	63 (7.9)	74 (8.9)	53 (7.0)	55 (7.2)	67 (8.3)	52 (6.9)	68 (8.4)	45 (6.3)	84 (9.8)	48 (6.5)	48 (6.5)	48 (6.5)	45 (6.3)	53 (7.0)	54 (7.1)	54 (7,1)	49 (6.6)	65 (8.1)	68 (8.4)	51 (6.8)	64 (8.0)	99 (11.2)	54 (7.1)
Fasting plasma glucose (mg dL^{-1})	240	111	167	159	179	139	190	249	208	172	150	156	186	142	218	166	156	132	163	212	163	168	187	226	209	86	186	285	181
HDL (mg dL^{-1})	65	54	82	43	55	65	57	45	70	45	51	59	46	54	-	41	59	45	61	55	58	58	51	53	50	72	53	68	-

Total cholesterol (mg dL^{-1})	230	134	205	135	162	203	172	146	171	125	155	165	142	120	-	120	125	101	139	125	175	149	154	137	144	163	152	161	-
LDL (mg dL^{-1})	111	66	87	79	95	118	105	58	98	65	95	93	96	58	-	71	59	45	69	60	108	79	84	73	73	83	83	75	-
Triglycerides (mg dL^{-1})	231	71	181	64	59	99	48	213	54	77	44	63	30	39	-	40	36	44	45	49	47	61	94	56	103	39	79	88	-
25- oH- vitamin D total (ng mL^{-1})	38	11	19	18	20	26	39	16	13	21	13	25	15	12	-	20	7	21	25	10	19	33	29	34	21	30	22	33	-
Urea (mg dL^{-1})	27	31	23	24	23	33	27	33	25	21	19	29	26	39	29	23	32	27	30	24	17	36	46	40	26	29	26	35	-
Creatinine (mg dL^{-1})	0.7	0.7	0.67	0.75	0.58	0.63	0.77	0.53	0.74	0.58	0.79	0.56	0.40	0.60	0.42	0.60	0.49	0.77	0.51	0.48	0.69	0.53	0.96	0.97	0.69	0.68	0.70	0.88	-
Albuminuria (mg L^{1})	12.2	7.3	11.4	3.1	6.3	1.7	4.3	5.3	21.3	9.0	17.6	7.2	3.9	3.7	12.1	3.2	5.6	6.9	3.7	3.2	1.2	1.2	7.1	10.7	45.8	126.8	9.5	1.6	-
Albumin/ creatinine Ratio (mg g^{-1} creatinine)	6.7	7.3	4.8	1.4	2.7	1.5	11.5	10.6	8.0	3.7	8.9	6.5	4.3	3.4	9.5	2.2	3.7	2.9	2.6	3.5	6.6	1.2	2.6	6.8	23.3	48.0	4.3	1.6	-
Time In Range (%)	29%	84%	72%	61%	49%	75%	46%	35%	65%	60%	43%	85%	48%	75%	14%	68%	74%	77%	67%	73%	63%	62%	70%	-	44%	69%	43%	15%	30%
Variation Coefficient (%)	41%	32%	30%	41%	51%	43%	43%	49%	34%	34%	38%	36%	46%	31%	36%	38%	30%	33%	37%	38%	35%	32%	33%	-	49%	39%	39%	38%	45%
Muscular atrophy (Present=1 Absent=0)	0	0	0	0	0	0	0	0	0	0	0	0	0	0	0	0	0	0	0	0	0	0	0	0	0	0	0	0	0
Ulcers(present=1 Absent=0)	0	0	0	0	0	0	0	0	0	0	0	0	0	0	0	0	0	0	0	0	0	0	0	0	0	0	0	0	0
Pateller reflex (normal =0 increased=1, decreased or absent =2)	0	0	0	0	0	0	0	0	2	0	2	0	0	0	0	0	2	0	0	2	0	0	0	0	0	0	0	0	0
Bicipital Relfex (normal =0 increased=1 decreased or absent =2)	0	0	0	0	0	0	0	0	0	0	0	0	0	0	0	0	0	0	0	0	0	0	0	0	0	0	0	0	0
Tricipital Reflex (normal =0 increased=1 decreased or absent=2)	0	0	0	0	0	0	0	0	0	0	0	0	0	0	0	0	0	0	0	0	0	0	0	0	0	0	0	0	0
Achilles reflex (normal =0	0	0	0	0	0	0	0	0	2	0	2	0	0	0	0	0	2	0	0	2	0	0	0	0	0	0	0	0	0

increased=1 decreased or absent =2)																													
Vibration (present =1 absent=0)	1	1	1	1	1	1	1	1	1	1	1	1	1	1	1	1	1	1	1	1	1	1	1	1	1	1	1	1	1
Cutaneous sensation (present=1 absent=0)	1	1	1	1	1	1	1	1	1	1	1	1	1	1	1	1	1	1	1	1	1	1	1	1	1	1	1	1	1
Muscular force-dorsiflexion (0-5)	5	5	5	5	5	5	5	5	5	5	5	5	5	5	5	5	5	5	5	5	5	5	5	5	5	5	5	5	5
Muscular force-plantar flexion (0-5)	5	5	5	5	5	5	5	5	5	5	5	5	5	5	5	5	5	5	5	5	5	5	5	5	5	5	5	5	5
Muscular force-knee flexion (0- 5)	5	5	5	5	5	5	5	5	5	5	5	5	5	5	5	5	5	5	5	5	5	5	5	5	5	5	5	5	5
Muscular force-knee extension (0-5)	5	5	5	5	5	5	5	5	5	5	5	5	5	5	5	5	5	5	5	5	5	5	5	5	5	5	5	5	5
Muscular force-elbow flexion (0-5)	5	5	5	5	5	5	5	5	5	5	5	5	5	5	5	5	5	5	5	5	5	5	5	5	5	5	5	5	5
Muscular force-elbow extension (0-5)	5	5	5	5	5	5	5	5	5	5	5	5	5	5	5	5	5	5	5	5	5	5	5	5	5	5	5	5	5
Burning sensation (present=1 absent=0)	0	0	0	0	0	0	0	0	0	0	0	0	0	0	0	0	0	0	0	0	0	0	0	0	0	0	0	0	0
Shock like sensation (present=1 absent=0)	0	0	0	0	0	0	0	0	0	0	0	0	0	0	0	0	0	0	0	0	0	0	0	0	0	0	0	0	0
Tingling (present=1 absent=0)	0	0	0	0	0	0	0	0	0	0	0	0	0	0	0	0	0	0	0	0	0	0	0	0	0	0	0	0	0
Numbness (present=1 absent=0)	0	0	0	0	0	0	0	0	0	0	0	0	0	0	0	0	1	0	0	0	0	0	0	0	0	0	0	0	0
Cramps (present= 1 absent=0)	0	0	0	0	0	0	0	0	0	0	0	0	0	0	0	0	0	0	0	0	0	0	0	0	0	0	0	0	0
Fatigue (present=1 absent=0)	0	0	0	0	0	0	0	0	0	0	0	0	0	0	0	0	0	0	0	0	0	0	0	0	0	0	0	0	0
Instability while walking (present=1 absent=0)	0	0	0	0	0	0	0	0	0	0	0	0	0	0	0	0	0	0	0	0	0	0	0	0	0	0	0	0	0
Hyperalgesia(present=1 absent=0)	0	0	0	0	0	0	0	0	0	0	0	0	0	0	0	0	0	0	0	0	0	0	0	0	0	0	0	0	0
Allodynia (present=1 absent=0)	0	0	0	0	0	0	0	0	0	0	0	0	0	0	0	0	0	0	0	0	0	0	0	0	0	0	0	0	0

Latency- left medianus motor (m s)	3.12	3.29	3.33	3.71	3.71	3.71	3.58	3.21	3.17	2.92	3.64	3.38	2.97	3.85	3.83	3.25	2.83	4.17	3.46	3.04	3.56	3.42	3.4	3.79	2.88	3.1	3.1	3.65	3.1
Amplitude-left medianus motor (m V)	9.5	8.1	7.4	11.4	6.5	8.2	7.8	6.4	10.0	11.0	11.8	6.3	5.4	11.5	7.2	8.5	9.0	9,6	6.0	10.7	5.5	6.3	8.9	8.6	7.5	12.8	8.3	7.9	7.2
Conduction velocity-left medianus motor (m s^{-1})	56.8	60.7	56.5	59.7	50.1	50.8	54.7	55.6	55.3	55.4	65.0	56.4	57.1	55.8	54.7	59.7	57.6	58.7	60.4	58.8	53.9	65.5	59.3	56.2	50.3	57.6	57.0	55.0	55.6
Latency-Right medianus motor (m s)	3.42	2.81	3.38	3.73	3.46	3.88	3.60	3.42	3.44	2.98	3.60	3.42	2.94	4.23	3.46	3.17	3.02	3.69	3.15	3.15	3.98	3.46	3.19	3.69	3.29	2.81	3.13	3.69	3.06
Amplitude-Right medianus motor (m V)	9.9	9.6	8.6	7.5	9.5	9.5	12.5	9.1	11.3	12.3	11.0	6.1	5.1	9.3	9.1	10.5	8.9	11.3	8.6	9.5	6.2	7.7	13.5	9.3	8.4	11.5	8.5	6.7	10.2
Conduction velocity-Right medianus motor (m s^{-1})	57.2	58.4	59.4	53.9	59.3	58.0	59.9	51.7	58.0	55.3	57.3	60.1	66.9	55.8	57.0	60.1	59.2	59.1	58.5	57.1	54.5	60.5	58.7	58	56.7	58.5	**58.0**	55.1	60.3
Latency-Left peroneus motor (m s)	5.17	3.25	4.29	4.25	4.46	4.9	4.79	3.33	4.25	3.9	4.06	3.91	3.65	4.4	4.6	4.1	3.17	4.33	5.17	4.27	3.16	3.98	3.83	4.69	4.06	3.99	3.83	3.81	3.65
Amplitude-left peroneus motor (m V)	5.2	7.1	3.7	5.8	4.0	6.8	8.2	6.7	6.8	3.0	5.3	3.9	1.46	3.5	4.9	4.5	5.4	6.2	3.3	5.5	4.0	5.0	13.1	7.2	5.3	4.6	6.2	7.0	9.3
Conduction velocity-Left peroneus motor (m s^{-1})	44	51.4	49.1	48.7	57.4	56.1	54.4	49.4	52.7	50.5	43.7	50.1	46.5	40.5	47.6	48.7	49.6	52.4	51.4	50.7	51.0	51.4	52.0	46.8	46.7	48.6	50.4	47.6	49.3
Latency-right peroneus motor (m s)	4.33	3.10	3.69	3.48	3.60	3.65	3.63	3.32	3.85	3.58	4.68	3.54	3.81	4.23	3.67	4.19	3.77	4.27	3.92	4.38	3.85	3.97	3.44	3.65	3.73	3.67	3.37	4.13	3.48
Amplitude-right peroneus motor (m V)	6.6	7.6	4.7	6.6	4.6	7.8	9.6	6.1	8.2	3.6	5.2	3.8	2.8	5.6	10.1	3.9	4.3	7.3	6.1	6.7	5.3	7.1	11.0	9.7	7.2	2.9	6.3	5.1	7.8
Condution velocity-right peroneus motor (m s^{-1})	43.7	53.3	47.0	47.7	49.7	46.9	53.5	53.6	50.3	47.6	47.9	47.0	52.5	40.4	44.4	47.0	54.7	48.9	48.6	50.9	48.3	51.5	50.6	44.1	45.8	47.7	50.0	47.0	51.3
Latency-left ulnaris motor (m s)	2.48	2.46	2.48	2.62	2.6	2.85	2.92	2.56	2.3	2.6	3.13	2.44	2.38	3.21	2.42	2.31	1.96	3.23	2.35	2.56	2.44	2.42	2.6	2.9	2.23	2.25	2.42	2.63	2.52
Amplitude-left ulnaris motor (m V)	8.9	9.7	8.3	8.6	7.0	9.9	10.6	8.1	7.7	6.8	9.7	10.3	7.4	7.7	7.5	8.7	8.9	9.0	7.0	9.1	7.7	7.3	10.9	10.5	7.9	9.6	9.0	11.3	6.7

Conduction velocity-left ulnaris motor (m s^{-1})	53.2	67.0	58.8	68.4	56.8	59.2	**60.89**	58.6	63.5	68.6	59.1	57.1	70.4	61.2	58.0	63.5	66.1	56.9	64.1	60.3	56.7	61.6	59.6	61.6	52.3	60.7	59.8	57.7	67.1
Latency-right ulnaris motor (m s)	2.73	2.38	2.69	2.52	2.83	2.38	3.23	2.71	2.69	2.44	3.25	2.67	2.46	3.25	2.58	2.65	2.06	3.04	2.38	2.65	2.9	2.56	3.21	2.52	2.48	2.27	2.44	2.6	2.5
Amplitude-right ulnaris motor (m V)	8.8	10.1	7.4	9.0	6.2	9.4	10.3	7.4	7.7	6.1	9.4	8.5	9.6	8.2	8.1	7.8	9.0	9.5	6.5	9.1	10.3	6.6	11.9	8.7	6.6	10.9	11.1	9.6	6.2
Conduction velocity-right ulnaris motor (m s^{-1})	65.0	65.7	56.0	68.6	56.9	56.0	57.1	53.6	**60.12**	63.3	56.1	65.3	70.9	55.7	54.5	63.8	60.4	51.3	66.7	60.3	62.3	61.7	64.5	56.7	51.3	60.4	59.0	59.3	61.0
Latency-left tibialis motor (m s)	4.98	4.13	4.09	3.25	3.87	3.63	4.58	4.62	3.44	3.82	3.16	4.35	3.92	4.32	3.99	3.7	3.68	5.23	4.44	3.92	5.04	4.51	3.65	4.08	3.43	3.75	4.13	4.85	4.76
Amplitude-left tibilais motor (m V)	7.8	12.2	6.3	17.3	6.0	9.7	11.5	9.5	10.2	5.8	11.1	9.7	10.1	9.1	13.7	11.1	16.4	11.1	15.0	9.8	16.8	14.7	23.1	9.5	10.6	11.4	9.3	9.8	11.8
Latency-left tibialis motor (m s)	4.79	3.33	4.14	4.58	3.83	3.75	4.9	4.17	4.96	3.54	5.13	3.92	3.23	4.9	4.45	3.32	3.73	4.96	4.94	4.24	4.08	3.7	4.46	3.62	4.35	4.54	4.63	3.92	4.73
Amplitude-Right tibialis motor (m V)	6.9	9.6	8.9	13.2	8.3	13.2	12.4	10.1	9.2	9.9	12.2	7.2	7.4	10.7	15.5	9.4	14.1	16.7	14.0	9.6	13.7	14.5	17.2	7.4	13.7	11.6	16.7	8.9	10.4
F minimal latency right (m s)	50.2	42.4	47.3	44.2	43.4	48.3	44.9	44.5	49.7	42.5	52.2	41.4	37.8	54.2	47.7	48.0	39.3	46.4	49.0	42.3	42.6	42.4	42.8	49.7	48.7	44.5	45.6	46.2	44.0
F minimal latency left (m s)	54.2	41.8	46.2	43.2	42.9	46.9	44.7	44.1	52.1	42.8	50.1	44.5	37.4	54.4	48.5	46.3	39.8	46.2	49.1	41.5	44.4	41.1	44.3	49.9	48.8	45.4	45.9	47.5	43.2
